# *In-vitro* Synergistic Effect of Metformin and Berberine on High Glucose-induced Lipogenesis

**DOI:** 10.22037/ijpr.2019.15085.12867

**Published:** 2019

**Authors:** Reyhaneh Babaei Khorzoughi, Fatemeh Namvarjah, Maryam Teimouri, Hossein Hosseini, Reza Meshkani

**Affiliations:** *Department of Clinical Biochemistry, Faculty of Medicine, Tehran University of Medical Sciences, Tehran, Iran.*

**Keywords:** Diabetes, Lipogenesis, Metformin, Berberine, Combination, NAFLD

## Abstract

Metformin and berberine have been reported to have lipid lowering effects. This study aims to investigate lipid lowering effects of berberine and Metformin, alone and in combination, in HepG2 cells to determine whether berberine and Metformin work synergistically and elucidate their mechanisms. HepG2 cells were treated with 33 mM glucose in the presence of various concentrations of berberine and Metformin, alone and in combination, for 24 h. The cytotoxic effects of these compounds were determined by MTT assay. Oil red O staining, triglyceride measurement, and gene expression analyses were performed to evaluate the effects of these compounds on hepatocytes lipogenesis. Berberine at doses 20 µM and 40 µM and Metformin at doses 1 mM and 2 mM reduced total lipid content and triglyceride level in HepG2 cells. Metformin (mM) and berberine (µM) at combination ratios of 2:40, 1:20, 0.5:10, and 0.25:5 exhibited a synergistic lipid-lowering effect on HepG2 cells. These ratios could significantly decrease total lipid content and triglyceride level in HepG2 cells. The lowest dose of the combination [Metformin (0.25 mM) and berberine (5 μM)] also synergistically reduced the expression of the FAS and SREBP-1c genes in HepG2 cells treated with high glucose. The combination of Metformin and berberine exerted synergistic lipid-lowering effects on HepG2 cells by reducing total lipid content, triglyceride level, and the expression of the genes involved in lipogenesis.

## Introduction

The increasing prevalence of type 2 diabetes (T2D) worldwide is one of the most serious and challenging health problems in the 21^st^ Century. The World Health Organization estimates that the number of people with diabetes will double by 2030 (1). Defects in insulin action (insulin resistance) and insulin secretion (β cell dysfunction) are two key players in the pathophysiology of T2D (2). Insulin resistance is characterized by an increased hepatic glucose production and a decreased glucose uptake in peripheral tissues (3). In the liver, insulin resistance is an important underlying cause of dyslipidemia in subjects with nonalcoholic fatty liver disease (NAFLD) and T2D (3). The dyslipidemia is characterized by increased plasma levels of triglyceride and small, dense low-density lipoproteins (sdLDL) and decreased level of HDL cholesterol (3). Insulin regulates hepatic very low density lipoprotein (VLDL) production, especially VLDL1, by affecting the rate of apoB synthesis and degradation and hepatic de novo lipogenesis. Insulin also regulates VLDL production by indirectly modulating free fatty acids (FFAs) flux from adipose tissue into the liver (4, 5). Under insulin resistant condition, hyperinsulinemia can activate sterol regulatory element binding protein -1c (SREBP-1), a major transcriptional regulator that induce key lipogenic enzymes such as fatty acid synthase (FAS) to promote lipogenesis in the liver (6). Furthermore, in insulin resistance state, the increased release of FFAs from adipose tissue results in the excessive lipid accumulation in hepatocytes leading to hepatic steatosis and inflammation. In this regard, any strategy to decrease lipid accumulation in the liver may provide a protective effect against NAFLD and T2D. Metformin, the most widely used anti-diabetic drug is an insulin-sensitizing agent that provides glycemic control, especially in obese individuals (7). Metformin has been reported to have the ability to reduce hepatic de novo lipogenesis (8). Metformin is typically required at higher doses for optimal effects (9). Moreover, this drug exhibits several side effects such as hypoglycemia, liver toxicity, lactic acidosis, and diarrhea (10). For many patients, Metformin monotherapy is insufficient to achieve glycemic targets, and therefore, additional therapies are advised (11). 

Recent evidence highlights the potential value of phytochemical abundantly found in some plants and common dietary preparations, in helping relieve clinical complications in subjects with diabetes and NAFLD. Phytochemicals have been proposed to be useful as an adjuvant therapy for their potential anti-diabetic, anti-obesity, anti-oxidant, anti-inflammatory, and lipid-lowering effects. Berberine, an isoquinoline alkaloid extracted from Coptis Chinesis has multiple pharmacological effects including anti-microbial, anti-inflammation, anti-cancer, anti-obesity, anti-diabetic, and anti-hyperlipidemia (12). Most investigations on anti-diabetic, anti-obesity, anti-oxidant, anti-inflammatory, and lipid-lowering effects of berberine were conducted with the individual compound. However, berberine has been proposed to be useful as an adjuvant therapy for their potential anti-diabetic, anti-obesity, anti-oxidant, anti-inflammatory, and lipid-lowering effects (13). The present study was designed to determine whether a blend of Metformin and berberine would act synergistically to control high glucose-induced lipogenesis *in-vitro*. The intention was to develop a formulation that uses a very low dose of Metformin in combination with berberine in order to lower the effective dose of Metformin required for management of diabetes and NAFLD. 

## Experimental

*Materials*

Dulbecco’s modified Eagle’s medium (DMEM), fetal bovine serum (FBS), and trypsin EDTA were purchased from Gibco (Gibco, Germany). Tissue culture flasks and disposable plastic were purchased from Greiner Bio-One (Frickenhausen, Germany). Metformin and berberine were purchased from Santa Cruz Biotechnology, Inc. (Dallas, Texas, United States). All other reagents and chemicals were from Sigma Aldrich.

*Cell culture and MTT assay*

HepG2 cells were purchased from the Iranian Biological Resource Center (IBRC). The cells were cultured at 37 °C (in an atmosphere of 5% CO2) in DMEM supplemented with 10% FBS, and 1% penicillin-streptomycin. In order to induce lipogenesis, HepG2 cells were stimulated with 33 mM D-glucose (HG) for 24 h. As an osmotic control, we used D-mannitol (27.5 mM mannitol) in normal glucose (NG) (5.5 mM glucose) treatments. Selection of the time and dose of glucose treatments were based on a previous published study (14). Cytotoxitocy of Metformin and berberine were determined by MTT assay. The cells were seeded in 96-well plates at 10000 cells/well in 0.1 mL of culture media and then incubated with different concentrations of the compounds. HepG2 cells were pretreated with Metformin and berberine alone or in combination for 2 h. 

*Oil red O staining*

To investigate whether berberine could potentiate the effect of Metformin on high glucose-induced lipogenesis in HepG2 cells, we evaluated total lipid content by oil red O staining. HepG2 cells were washed in cold PBS and then fixed with 4% paraformaldehyde solution for 30 min at room temperature. After washing with distilled water and incubating for 10 min in 60% isopropanol, the cells were stained for 20 min with fresh oil red O working solution (300 mg of oil red O powder and adding this to 100 mL of 99% isopropanol). The stained cells were washed thoroughly with distilled water and then were imaged using an Olympus camera mounted on an Olympus upright microscope. In order to quantify, 250 μL DMSO was added to the dried plates and then the optical density was measured at 510 nm. The protein concentration was determined by the bicinchoninic acid assay (BCA) method. The results were normalized against total protein.

*Determination of triglyceride*

The treated cells were washed twice with PBS, and then lysed by RIPA buffer (50 mM Tris–HCl, pH 7.4, 1% Triton X-100, and 0.2% sodium deoxycholate, 0.2% SDS, 1 mM Na-EDTA, and 1 mM PMSF) supplemented with protease inhibitor cocktail buffer for 30 min. After centrifugation at 12000 × g for 20 min at 4 °C, the supernatant was transferred to a new tube. Intracellular triglyceride content was measured with the Biovision triglyceride quantification Colorimetric/Fluorimetric kit (Biovision Inc, U.S) according to the manufacturer′s instructions. The protein concentration was determined by the BCA method. The results were normalized against total protein level.

*Gene expression analysis*

Total RNA was extracted using GeneAll RibospinTM kit (GeneAll Biotechnology, South Korea). Complementary DNA (cDNA) was reverse transcribed using a RevertAid First Strand cDNA Synthesis Kit (Thermo Fisher Scientific, U.S). Gene expression was quantified using specific primers for fatty acid synthase (FAS), and sterol regulatory element-binding protein 1c (SREBP-1c) using SYBR Green RealQ Plus 2x Master Mix Green (Ampliqon) on Applied Biosystems Real-Time PCR (Thermo Fisher Scientific, U.S). The sequences of the primers used in the study are in Table S1 (supplementary file). The levels of the target gene transcripts were normalized relative to β-actin. The gene expression in fold-change was calculated by delta-delta Ct method.

*Statistical analyses*

All statistical analyses were performed using IBM SPSS Statistics 22 (IBM, USA). Comparisons among all groups were performed with unpaired student’s t-test or one-way analysis of variance (ANOVA) test. If statistical significance was found, the Tukey post hoc test was performed. Values of *P* < 0.05 were considered statistically significant. Results are expressed as mean ± SEM of three independent experiments. Compusyn software was used to analyze the synergistic effect of Metformin and berberine. Combination index (CI) values were generated by the Compusyn software. CI < 1, CI = 1, and CI > 1 represent synergism, additivity, or antagonism, respectively.

## Results

*Metformin and berberine decrease lipid content in HepG2 cells*

**Figure 1:**
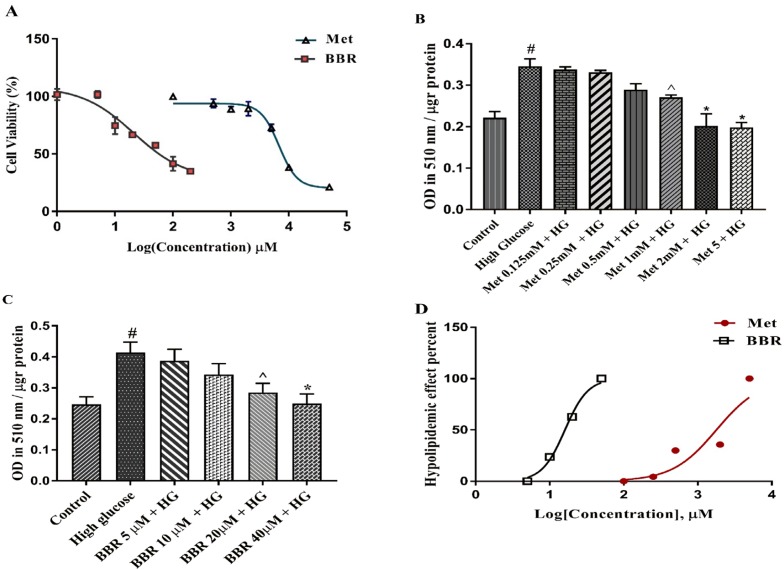
The cytotoxicity and lipid lowering effect of Metformin and berberine on HepG2 cells: (A) Cell growth activity was measured by MTT assay. IC50 of Metformin and berberine were 9.819 mM 65.83 μM, respectively. (B-C) Total lipid content of the cells treated by different concentrations of Metformin and berberine alone. Total lipid content was evaluated by Oil red O staining method. (D) EC50 for Metformin and berberine treatments were 1.786 mM and 16.07 μM, respectively. Represented data are from three independent experiments and are means ± SEM. #: Control *vs. *High glucose, *: Treatments *vs. *High Glucose with *P *< 0.001, ^: Treatments *vs. *High Glucose with *P *< 0.05. Control: D-mannitol (27.5 mM mannitol) in normal glucose (NG) (5.5 mM glucose); HG: High (33 mM glucose); Met: Metformin; BBR: Berberine

We first evaluated the cytotoxic effects of the selected compounds on HepG2 cells using MTT assay. The cells were treated with various concentrations of Metformin and berberine for 24 h. As shown in Figure 1A, IC50s (half maximal inhibitory concentration) the values obtained from our results were 9.819 mM for Metformin and 65.83 μM for berberine. We then induced lipogenesis by treating HepG2 cells with 33 mM glucose (HG). As shown in Figures 1B-1C, HG significantly increased total lipid content compared to the control (*P* < 0.0001). Metformin and berberine individually could significantly decrease HG-induced total lipid content in the HepG2 cells. The EC50 (half maximal effective concentration) from our Oil red O staining results were 1.786 mM for Metformin and 16.07 μM for berberine (Figure 1D). The EC50, the concentration of a drug that gives half-maximal response, was calculated by GraphPad Prism software.

*Combination of Metformin and berberine is more potent than the individual components in reducing total lipid in HepG2 cells*

**Figure 2:**
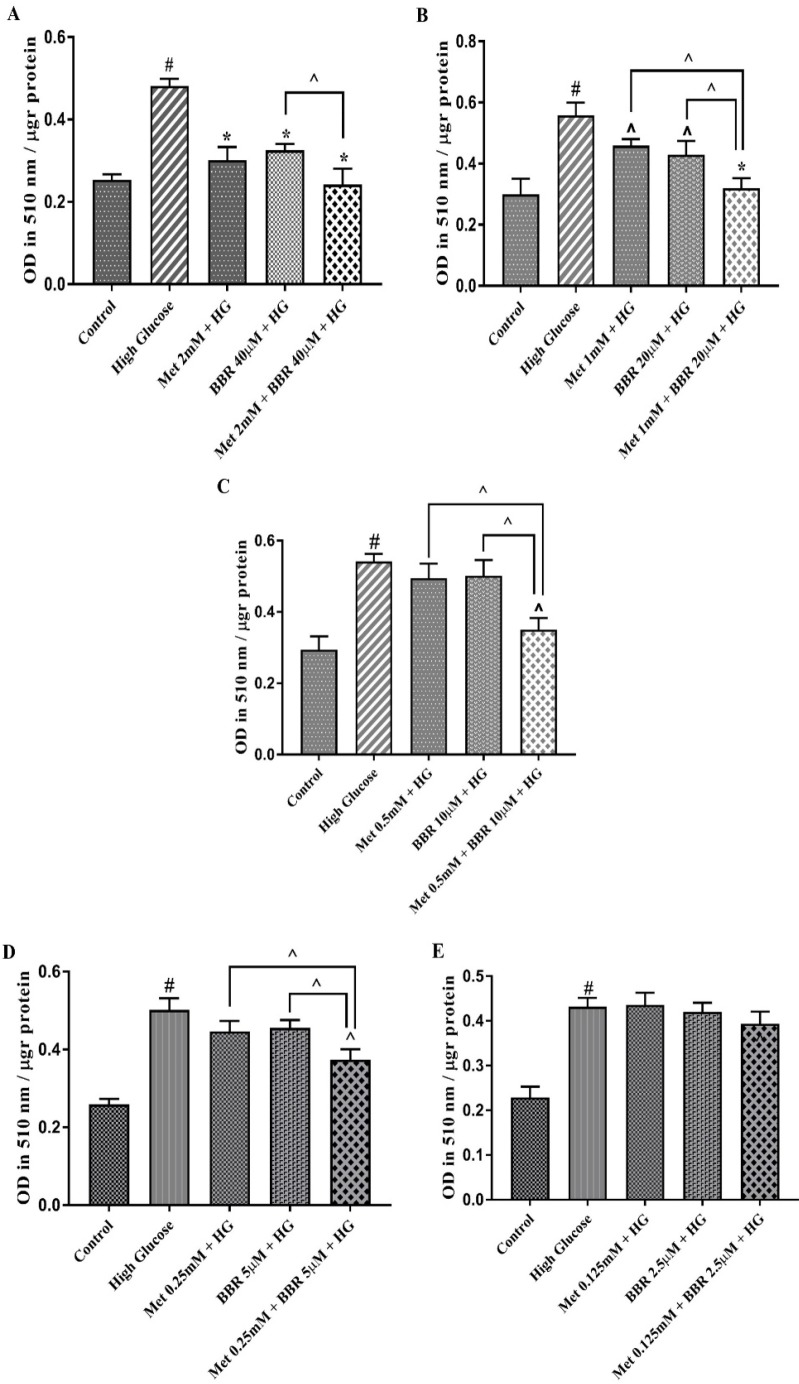
Total lipid content of the cells treated by Met, BBR, and Met + BBR. HepG2 cells were treated with a constant molar ration of 50:1 Metformin to berberine. Total lipid content was evaluated by Oil Red O staining method. (A) Metformin (mM) to berberine (µM) of 2:40, (B) Metformin (mM) to berberine (µM) of 1:20, (C) Metformin (mM) to berberine (µM) of 0.5:10, (D) Metformin (mM) to berberine (µM) of 0.25:5, (E) Metformin (mM) to berberine (µM) of 0.125:2.5. Represented data are from three independent experiments and are means ± SEM. #: Control *vs. *High glucose, *: Treatments *vs. *High Glucose with *P *< 0.001, ^: Treatments *vs. *High Glucose with *P *< 0.05. Control: D-mannitol (27.5 mM mannitol) in normal glucose (NG) (5.5 mM glucose); HG: High Glucose (33 mM glucose); Met: Metformin, and BBR: Berberine. ^: Metformin and berberine alone *vs*. combination treatment (Met + BBR)

To investigate whether berberine could potent the effect of Metformin, we evaluated HG-induced lipogenesis by oil red O staining in the HepG2 cells treated with the various concentrations of combined Metformin and berberine in a fixed molar ratio (50:1) (Figure S1). A mixture of Metformin (2 mM), and berberine (40 µM) reduced hepatocytes lipid content to control level that was significantly more potent than what was observed for the individual compounds. Reducing the dosage of both compounds in a fixed molar ratio revealed that the combination of Metformin (mM): berberine (µM) at concentrations of 1:20, 0.5:10, and 0.25:5 have a synergistic lipid lowering effect (CI = 0.26, CI = 0.34 and CI = 0.44, respectively) (Figures 2A-2E). All these ratios reduced HG-induced lipid accumulation in HepG2 cells. Our Data demonstrated that the combination of Metformin (mM): berberine (µM) at concentration of 0.125:2.5 had no significant lipid lowering effect in hepatocytes. 

*Combination of Metformin and berberine synergistically decrease intracellular triglyceride level*

**Figure 3:**
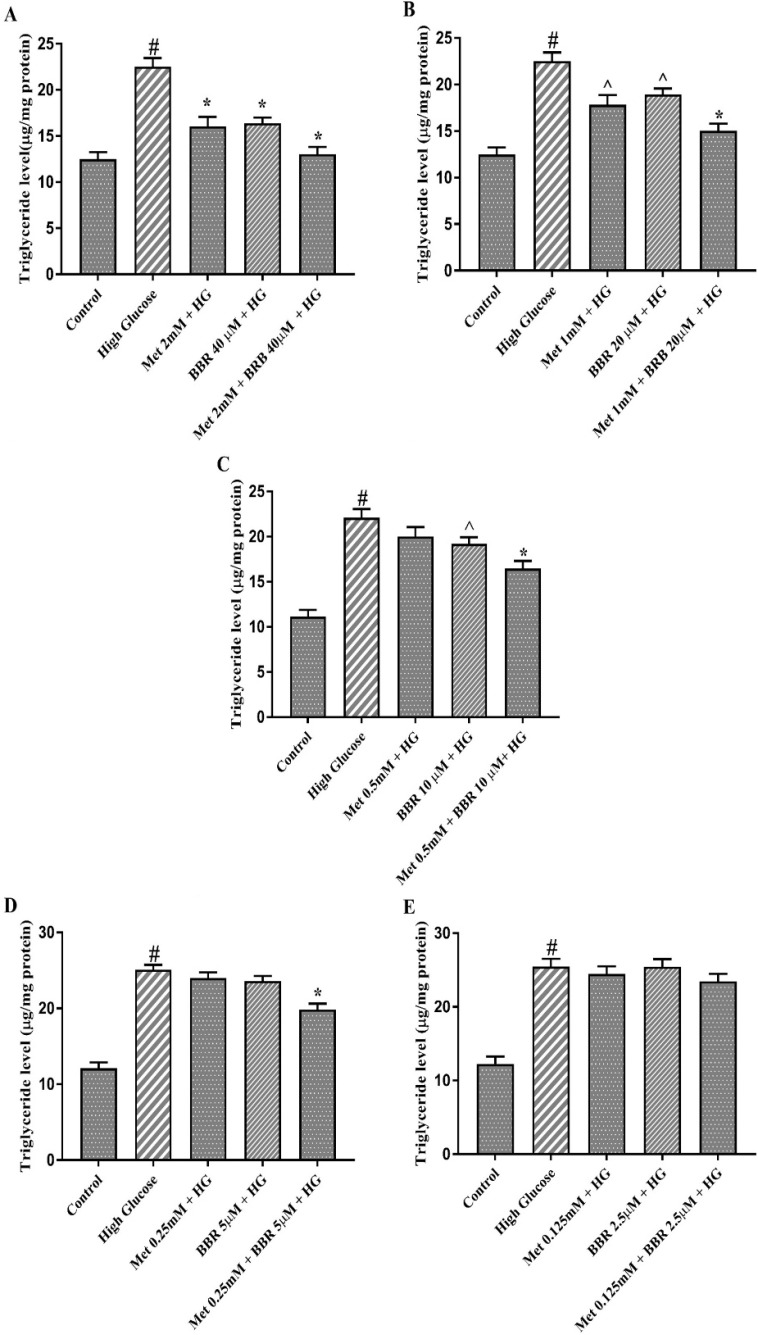
Triglyceride level of the cells treated by Met, BBR, and Met + BBR. HepG2 cells were treated with a constant molar ration of 50:1 Metformin to berberine. (A) Metformin (mM) to berberine (µM) of 2:40, (B) Metformin (mM) to berberine (µM) of 1:20, (C) Metformin (mM) to berberine (µM) of 0.5:10, (D) Metformin (mM) to berberine (µM) of 0.25:5, (E) Metformin (mM) to berberine (µM) of 0.125:2.5. Represented data are from three independent experiments and are means ± SEM. #: Control *vs. *High glucose, *: Treatments *vs. *High Glucose with *P *< 0.001, ^: Treatments *vs. *High Glucose with *P *< 0.05. Control: D-mannitol (27.5 mM mannitol) in normal glucose (NG) (5.5 mM glucose), HG: High Glucose (33 mM glucose), Met: Metformin, and BBR: Berberine

In order to confirm above data, we measured triglyceride level in HepG2 cells. Combination of Metformin (mM) and berberine (μM) at ratios of 2:40 and 1:20, synergistically decreased triglyceride level in HepG2 cells treated with HG (CI = 0.16, and CI = 0.26, respectively) (Figures 3A and 3B). While Metformin alone at 0.5 and 0.25 mM had no a significant effect on triglyceride level, a synergistic effect (CI = 0.34) was found when Metformin (mM) and berberine (μM) were combined at doses of 0.5:10 and 0.25:5 (Figures 3C and 3D). The reducing hepatocyte triglyceride level was unaltered in the presence of low doses of Metformin (0.125 mM) or BBR (2.5 μM) alone or in combination (Figure 3E). Taken together, these results suggest that the combination of Metformin and berberine exerted synergistic lipid lowering effect on HepG2 cells.

*Combination of Met and BBR suppressed SREBP-1c and FAS expression in HepG2 cells*

**Figure 4:**
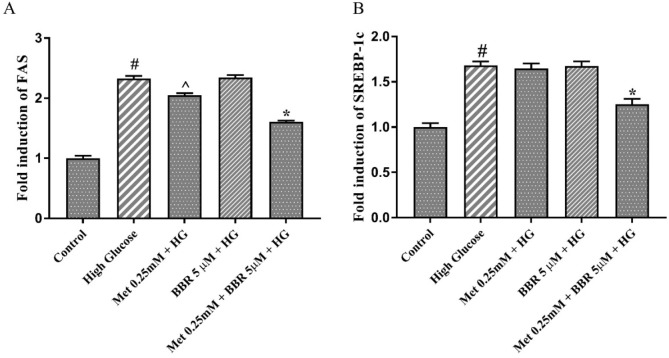
The expression of the key lipogenic genes, SREBP1c and FAS in the presence of Met, BBR, and Met+BBR. Represented data are from three independent experiments and are means ± SEM (n = 3). #: Control *vs. *High glucose, *: Treatments *vs. *High Glucose with *P *< 0.001, ^: Treatments *vs. *High Glucose with *P *< 0.05. Control: D-mannitol (27.5 mM mannitol) in normal glucose (NG) (5.5 mM glucose); HG: High (33 mM glucose); Met: Metformin; BBR: Berberine

In order to investigate the molecular mechanism underlying the lipid lowering effect of the combination of Metformin and berberine, we measured the expression level of the genes involved in lipogenesis such as FAS and SREBP-1c. For this purpose, we selected the lowest dosage of the combination [Metformin (0.25 mM) and berberine (5 μM)] that showed a synergistic lipid lowering effect. The results of real-time PCR indicated that HG increased the expression of SREBP-1c, and FAS genes compared to the control (approximately 2 fold change) (Figures 4A and 4B). When HepG2 cells were treated with 0.25 mM Metformin alone, a partial reduction of FAS expression (17%) and no significant effect on SREBP1-c expression were observed. By contrast, combining 0.25 mM Metformin with 5 µM berberine caused a significant reduction of SREBP-1c (25.5%), and FAS (31%) expression in HG-treated HepG2 cells (Figures 4A and 4B).

## Discussion

Increased de novo lipogenesis has been associated with NAFLD (15), insulin resistance (16, 17), diabetes (18), and obesity (19). Therefore, any strategies to reduce de novo lipogenesis in the liver might be beneficial in the treatment of NAFLD and T2D. Metformin, the most common antidiabetic agent, has been used as a glucose and lipid-lowering drug (20). Lin *et al.* have shown that Metformin causes the improvement of fatty liver in obese mice (21). Other animal model studies confirm the therapeutic effects of Metformin on fatty liver and insulin resistant (22-24). Human studies have also provided the evidence that Metformin consumption for 48 weeks can lead to improvement in histological abnormalities in nonalcoholic steatohepatitis (25). A systematic review and meta-analysis also indicated that Metformin improves liver function, homeostasis model assessment-insulin resistance (HOMA-IR), and body mass index to some extent in NAFLD patients (26). A daily oral dose of 2000–2500 mg/day is typically required for optimal effect of Metformin (9). At higher doses of Metformin, some patients report gastrointestinal symptoms, including diarrhea, cramps, nausea, and vomiting (27). Therefore, finding strategies to reduce the dose of Metformin required without compromising its efficacy is a useful approach for managing and reducing adverse events. 

Berberine is also able to reduce fat accumulation *in-vitro* and *in-vivo* (28, 29). A recent study by Zhao *et al.* has demonstrated that berberine inhibits hepatic lipogenesis through weight loss (30). The lipid lowering effect of berberine have been shown in HepG2 human hepatoma cells (31). Furthermore, the lipid lowering effect of berberine is comparable to those conventional lipid drugs but with low toxicity. Metformin and berberine share many features in actions despite different structure and both could be excellent drugs in treating T2D, obesity, cardiac diseases, tumor, as well as inflammation (32). Therefore, given the similarities of berberine and Metformin functions, the present study was designed to determine whether berberine would act synergistically with Metformin to decrease lipid accumulation in HepG2 cells. We first demonstrated that Metformin at doses 1 mM and 2 mM and berberine at doses 20 μM and 40 μM could reduce lipid accumulation in hepatocytes. After obtaining the dose-response of the individual components, we proceed to use the Oil red O staining method to discover a new combination that is more potent than the EC50 concentrations of any individual compound. In fact, we wanted to check the possibility of producing a mixture using two compounds at reduced concentrations but higher potency on lipid accumulation in HepG2 cells. This will allow us to use lower drug dosages, thereby reducing the associated costs and side effects. Our finding demonstrated that berberine could potentiate the lipid-lowering effect of Metformin on HepG2 cells and this effect appears to be related to the regulation of de novo lipogenesis pathway. 

Combining Metformin with berberine at a unique constant molar ratio (Metformin: berberine) 50:1 yielded a synergistic lipid lowering effect in HepG2 cells. We observed that the combination of Metformin (mM) and berberine (µM) at higher concentrations such as 2:40, 1:20, and 0.5:10 could significantly and synergistically reduce lipid accumulation and triglyceride level in HepG2 cells. Importantly, we could observe a synergistic lipid lowering effect for Metformin (mM) and berberine (µM) at concentration 0.25:5, while individual compounds at these concentrations did not exert lipid lowering effect in hepatocytes. The concentrations of Metformin (0.25 mM) and berberine (5 µM) in this experiment were less than EC50 of Metformin (1.78 mM) and berberine (16.07 µM). Taken together, these findings suggest that very low concentrations of Metformin and berberine synergistically could reduce lipid accumulation in hepatocytes. To the best of our knowledge, this is the first study to demonstrate that berberine and Metformin have synergistic lipid lowering effects in HepG2 cells. 

Cell culture and animal model experiments have shown that high glucose induces lipogenesis through upregulation of lipogenic genes including SREBP1-c and FAS (33-35). Similar to these findings, our data also demonstrated the ability of high glucose to induce lipogenic gene expression in HepG2 cells. It has been demonstrated that Metformin (36, 37) and berberine (38, 39) exert their inhibitory effects on lipogenesis through downregulation of SREBP-1s and FAS genes. Consistent with these observations, our data suggest that the combination of Metformin and berberine significantly reduce the expression of FAS and SREBP-1C in HepG2 cells. These findings suggest that the inhibitory effect of the combination of Metformin and berberin on lipogeneisis might be mediated through the supression of the expression of lipogenic genes. However, further mechanism studies should be conducted to clarify the effects of these compounds on the other potential signaling pathways regulating lipogenesis. In conclusion, this study demonstrated that the combination of Metformin and berberine exerted synergistic lipid-lowering effects on HepG2 cells by reducing total lipid content, triglyceride level, and the expression of genes involved in lipogenesis. Therefore, this combination could be a useful strategy to lower Metformin doses in treatment of T2D and NAFLD. However, further *in-vivo* studies are required to confirm the data of this *in-vitro* study.
